# Sample Size Calculation Guide - Part 5: How to calculate the sample size for a superiority clinical trial

**DOI:** 10.22114/ajem.v0i0.255

**Published:** 2019-08-29

**Authors:** Ahmed Negida, Nadien Khaled Fahim, Yasmin Negida, Hussien Ahmed

**Affiliations:** 1. Faculty of Medicine, Zagazig University, Zagazig, El-Sharkia, Egypt.; 2. Neurosurgery Department, School of Medicine, Bahçeşehir University, Istanbul, Turkey.; 3. Clinical Program, Faculty of Pharmacy, Zagazig University, Zagazig, Egypt.

## Introduction

In the previous educational articles, we explained how to calculate the sample size for a rate or a single proportion, for an independent cohort study, for an independent case-control study, and for a diagnostic test accuracy study (1–4). In this article, we explain how to calculate the sample size for a superiority clinical trial.

## When to use the sample size calculation procedure of superiority clinical trial

The methods explained hereafter should be used in the case that the superiority clinical trial is comparing two interventions with the endpoint being continuous data expressed as the mean difference.

## Requirements

1) Expected effect size (ES)
2) Type of clinical trial: Cross over or parallel
3) Allocation ratio between the experimental and control groups
4) Statistical power
5) Alpha

The expected ES differs according to the type of outcome measure. In case of continuous measures, the required ES will be the expected mean difference between the two arms and the standard deviation (SD) of the mean difference. In the case of binary outcomes (events as death or remission), the required ES will be the expected rate of event in each group.

## Calculation steps

1) Open SampSize application on your mobile
2) Select superiority from the type of trial (superiority, non-inferiority, or equivalence)
3) Select parallel for the design of the study (parallel or cross over)
4) Select normal for the type of outcome data (normal or binary)
5) Put the data into space and click “calculate.”

## Case study of subthalamic versus pallidal deep brain stimulation for patients with Parkinson’s disease

Assume that we are conducting a randomized controlled trial to compare the subthalamic (STN) and pallidal (GPi) deep brain stimulation (DBS) for patients with Parkinson’s disease. The primary outcome measure of this study is the improvement in motor function measured by the unified Parkinson’s disease rating scale (UPDRS-III). The most recent report comparing the two targets was published by Odekerken et al. (5) where the STN DBS and GPi DBS resulted in 20.3 and 11.4 point-improvements on the UPDRS-III, respectively. The SD of the motor functions (UPDRS-III) of two groups at baseline were 13.5 and 15.5, respectively.

## Case solution

First, we determine the requirements

- Expected mean difference between the two arms: 20.3 – 11.4 = 8.9
- $\text{SD}=\sqrt{\mathrm{SD}.{\mathrm{experimental}}^{2}+\mathrm{SD}.{\mathrm{control}}^{2}}=20$
- Type of clinical trial: parallel
- Allocation ratio between the experimental and control groups: 1
- Statistical power: 90%
- Alpha: 5%

**Figure 1:**
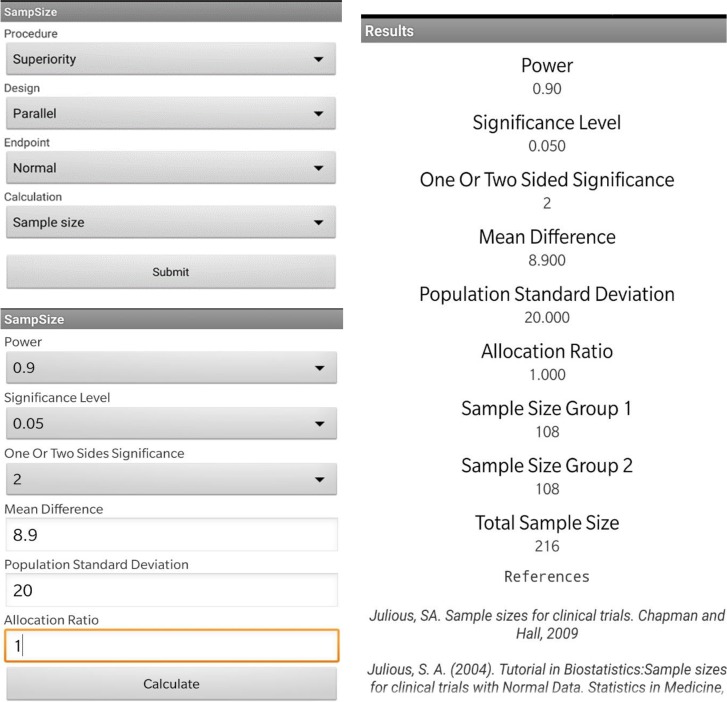
Calculating the sample size for a superiority clinical trial using an application

Second, we run the calculations as shown in figure 1. The results show that a minimum sample size of 216 patients (n=108 per group) will be required for this randomized controlled trial.
